# Evidence for human-centric in-vehicle lighting: part 3—Illumination preferences based on subjective ratings, eye-tracking behavior, and EEG features

**DOI:** 10.3389/fnhum.2023.1248824

**Published:** 2023-10-02

**Authors:** Christopher Weirich, Yandan Lin, Tran Quoc Khanh

**Affiliations:** ^1^Department of Illuminating Engineering and Light Sources, School of Information Science and Technology, Fudan University, Shanghai, China; ^2^Laboratory of Adaptive Lighting Systems and Visual Processing, Department of Electrical Engineering and Information Technology, Technical University of Darmstadt, Darmstadt, Germany

**Keywords:** in-vehicle lighting, lighting scene preference, electroencephalogram (EEG), cortical signal features, neuroaesthetics

## Abstract

Within this third part of our mini-series, searching for the best and worst automotive in-vehicle lighting settings, we aim to extend our previous finding about white light illumination preferences by adding local cortical area activity as one key indicator. Frontal electrical potential asymmetry, measured using an electroencephalogram (EEG), is a highly correlated index for identifying positive and negative emotional behavior, primarily in the alpha band. It is rarely understood to what extent this observation can be applied to the evaluation of subjective preference or dislike based on luminaire variations in hue, chroma, and lightness. Within a controlled laboratory study, we investigated eight study participants who answered this question after they were shown highly immersive 360° image renderings. By so doing, we first subjectively defined, based on four different external driving scenes varying in location and time settings, the best and worst luminaire settings by changing six unlabeled luminaire sliders. Emotional feedback was collected based on semantic differentials and an emotion wheel. Furthermore, we recorded 120 Hz gaze data to identify the most important in-vehicle area of interest during the luminaire adaptation process. In the second study session, we recorded EEG data during a binocular observation task of repeated images arbitrarily paired by previously defined best and worst lighting settings and separated between all four driving scenes. Results from gaze data showed that the central vehicle windows with the left-side orientated colorful in-vehicle fruit table were both significantly longer fixed than other image areas. Furthermore, the previously identified cortical EEG feature describing the maximum power spectral density could successfully separate positive and negative luminaire settings based only on cortical activity. Within the four driving scenes, two external monotonous scenes followed trendlines defined by highly emotionally correlated images. More interesting external scenes contradicted this trend, suggesting an external emotional bias stronger than the emotional changes created by luminaires. Therefore, we successfully extended our model to define the best and worst in-vehicle lighting with cortical features by touching the field of neuroaesthetics.

## 1. Review of the mini-series and study motivation

**Figure 1:**
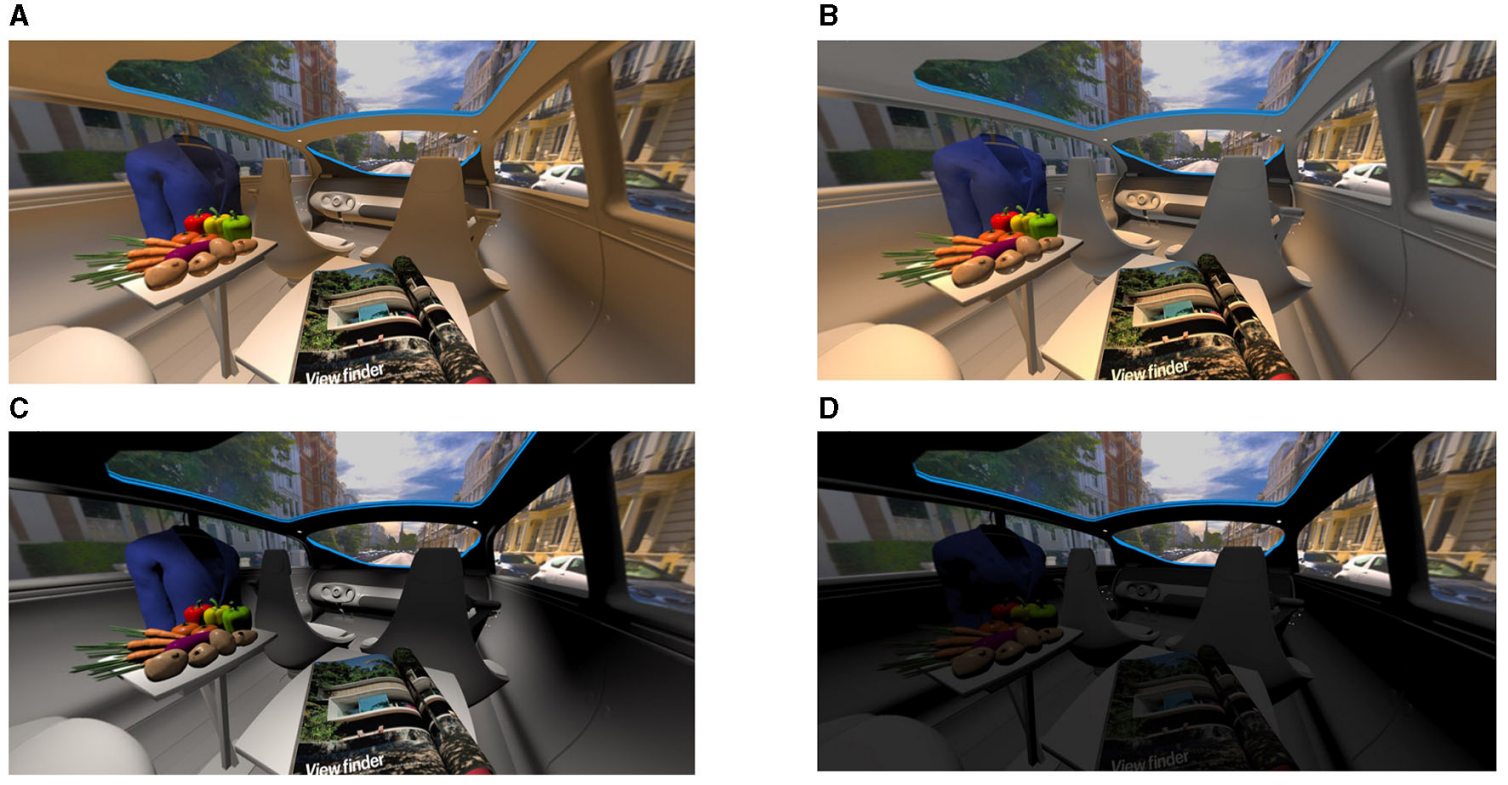
**(A, B)** Lighting settings for good in-vehicle illumination, named L6 and L7. **(C, D)** An example of a bad lighting setting, labeled L4 and L8. **(A–D)** Study results from our previous research (Weirich et al., 2022b).

In the coming years, personal individual driving will be progressively replaced by autonomous or self-driving vehicles, called robocars. Within this context, we investigated in part 1+2 of this mini-series the role of in-vehicle lighting personalized for vehicle occupants. Part 1 focused on general preferences for colors, positions, dynamics, and emotion relations evaluated globally between study participants from China and Europe with Likert-like questionnaires in a signaling context (Weirich et al., 2022a). In the study, we found a strong cultural color preference and color emotion dependency, especially in the level of attention, which is important for the driving context. Color emotion or mood dependency refers to the observation that different mono- and polychromatic light stimuli are connected to different emotional associations; for example, lower hue angles with red and yellow are connected more to the feeling of attention. This effect was valid for the European group but missing for study participants from China. Part 2 extended these findings to the context of general white light illumination (Weirich et al., 2022b). By displaying images as a 360° scene, we varied the correlation of color temperature (CCT), light distribution, and brightness settings, which were applied to four different driving scenes. These scenes varied in the time and location domains. To maintain a high level of variation, we decided to display one scene during a sunny day while driving in a city. The second scene showed a dim light narrow forest environment. In contrast, the third scene showed a wider, bright greenish countryside view. Finally, driving scene four occurred in Shanghai during the night, with colorful illuminated high-rise buildings. Within these settings, only a combination of focused and wider light cones with a mix of lower and higher CCTs performed better than all other light settings, and we named the light setting L6/L7; compare Figures 1A, B. This means that only a mix of lower and higher CCTs combined reached the highest ranking measured by psychological attributes. The in-vehicle scene was perceived within the Chinese and European groups with a higher level of satisfaction, value, and modernity. Furthermore, the scene itself was rated as more interesting with a higher level of spatial feeling and a brighter impression. By applying image transformation to the perceptional image spaces of IPT (Ebner and Fairchild, 1998) and CIE CAM16 (Li et al., 2017), we found, especially in the dimension of chroma, a strong correlation with the changes of the outer driving scene, which partially followed Hunt (1977). Furthermore, in the dimension of lightness and hue, for the best lighting setting, no lightness or hue shift should be observed between all four abovementioned external driving scenes and the in-vehicle cocoon. That means we were able to draft the first development guidelines for light technical engineers, but we lacked an understanding of the root cause. By presenting two representative examples, comparing Figures 1A, B for a good illumination and Figures 1C, D for a bad setting, we, as observers, are directly able to judge the preferred setting without hesitation. This clear decision-making process based on in-vehicle lighting variations is the exact target that must be understood within this presented part 3. Therefore, in the present study, we are not targeting a best or worst setting. Rather, we focus on subjective cortical decision mechanisms, which are involved based on preference or dislike in the context of in-vehicle lighting.

## 2. Introduction

To gain a better understanding of the cortical mechanisms responsible for defining a preferred or disliked luminaire setting, in this study, we focused on the combination of subjective emotional ratings and objective gaze data. Furthermore, we evaluated potential changes in an event-related potential (ERP) study, which is able to evoke emotional cortical electrical potentials measured using an electroencephalogram (EEG). The basic mechanisms of ERP studies are described in the literature (Husain, 2017; Lotte et al., 2018; Weirich et al., 2023). In short, during a time-locked presentation of repeated stimuli (in this study, we focus only on visual stimuli), it is possible to keep signals correlated with brain activity and, therefore, eliminate other signal activities, based only on EEG recordings. By repeating the same stimulus, the level of noise can, therefore, be reduced by the square root of the repetitions (Collura, 2000).

The significant time window of ERPs, which consist of emotional information, is ~300–500 ms (Righi et al., 2017) or up to 900–1,000 ms (Hajcak and Olvet, 2008) after stimulus onset. The observed signal peak is also called late positive potential (LPP). These potential changes could also be observed if the stimulus presentation was only 120 ms. Furthermore, the LPP between the left and right central electrode positions was observed around 300 ms after stimulus onset (Schupp et al., 2004). That means a delay time of ~180 ms might be observed between the stimulus and emotional reaction, which is nearly double compared to the P100 peak evoked by visual stimuli (Odom et al., 2016). For positive and negative emotions, the frontal alpha asymmetry (FAA), a power difference between the front right and front left hemispheres in the alpha waves between 7.5 and 12 Hz, is a very highly reliable signal indicator, as identified in a recent review (Byrne et al., 2022) and previously thoroughly researched (Ahern and Schwartz, 1985). Additionally, in the field of aircraft cabin preference, lower alpha band power was associated with lower preference and vice-versa, suggesting again a stronger alpha band responsibility (Ricci et al., 2022). Moreover, there were no congruent results between the identified preferences and final purchase behavior (Byrne et al., 2022). The authors reviewed that either there was a lack of statistical power or opposite findings, which led to the conclusion that there are no correlations between preferences and purchase behavior pertaining to the resulting decision-making process, measurable in the time domain by the LPP as an ERP component or in the frequency domain measurable by FAA. However, in a willingness-to-pay decision task, higher frontal asymmetric frequency band activities in the beta and gamma bands were strongly task-correlated compared with single alpha activity change (Ramsøy et al., 2018). That means it is still under debate which band or specific cortical indicator is finally responsible for a decision-making process based on connected emotions.

In the present study, we aim to enlighten this topic by applying a correlation study between subjective preferences and brain activity in the in-vehicle lighting context, which can be regarded as an interim stimuli strength comparing previous study settings with clear stimuli to achieve a strong happy–fear emotional change (Ahern and Schwartz, 1985). Furthermore, we want to understand whether these subjective lighting-based preferences can be decoded by cortical signal analysis.

Second, based on the above review of our mini-series, the understanding should be enhanced to categorize a presented in-vehicle lighting scene as good or bad. To achieve this, which scene detail is responsible for such a decision-making process will be investigated. According to the literature, especially the centrally located picture areas are first perceived. This effect is called a “central bias” (Tatler, 2007). However, whether this effect is more biased to the right or left side of in-vehicle tables will also be investigated, as shown in Figure 1. In such a scene, the level of visual attention per scene object might depend on differences between neighboring scene elements (Oyekoya and Stentiford, 2003). During the scanning process by the human visual system, alternating fast jumps—named saccades—and longer stops—called fixations—are executed. Information is primarily processed during the fixations. The fixation can start from 10 ms and last for several seconds (Holmqvist and Andersson, 2017). However, the scanning paths created by saccades and, therefore, the image location combined with the duration of fixations recorded by eye-tracking systems can provide insights into visual attention strategies. During a face preference study, participants should select which of the two presented faces they prefer. Initially, gaze data showed a balanced distribution between both images that finally shifted to the preferred face, named the “gaze cascade” effect (Shimojo et al., 2003). The authors of the study concluded that gaze plays a major role in preference decisions. This means that people tend to like what they look at and tend to look longer at objects that they like. In both cases, subjective value increased as reviewed recently (Wedel et al., 2023).

Therefore, this study combined approaches measuring cortical signal activity and gaze data, targeting a deeper understanding of in-vehicle lighting preferences. To this end, we formulated our research question as follows:

Q1: Are there specific objects available that are positioned in an in-vehicle lighting scene and are strongly connected to a prefer or dislike evaluation result?
Q2: How are the subjective preferences for in-vehicle lighting scenes coded in cortical signals?

## 3. Materials and methods

We separated our study design into two parts: First, we invited the study participants to define their most preferred and most disliked illumination settings, which are named in this study as good and bad lighting. For that, we created a highly immersive 360° image scene and presented it on a screen with six unlabeled luminaire sliders. Furthermore, we presented subjective scales as semantic differentials and as a five-step Likert-like emotion wheel to quantify the level of good or bad lighting. During the complete study period, we recorded gaze data to define special areas of interest that are connected to the preference decision-making process.

Second, we paired the abovementioned positive and negative in-vehicle lighting settings and presented them in a binocular image repetition study. In parallel, we recorded event-related cortical potentials using EEG. Finally, we extracted specific EEG signal features and compared them with event-related potentials that are highly correlated with strong positive and negative stimuli. Both sessions are explained in detail in the following Sections 3.1 and 3.2.

This study design was approved by the Ethical Committee of Fudan University, Approvement Number FE23073R. Furthermore, all eight participants gave written permission for attendance. They were also informed about possible study risks and had the possibility of quitting the study without a declaration.

### 3.1. Session 1: in-vehicle lighting preferences

**Figure 2:**
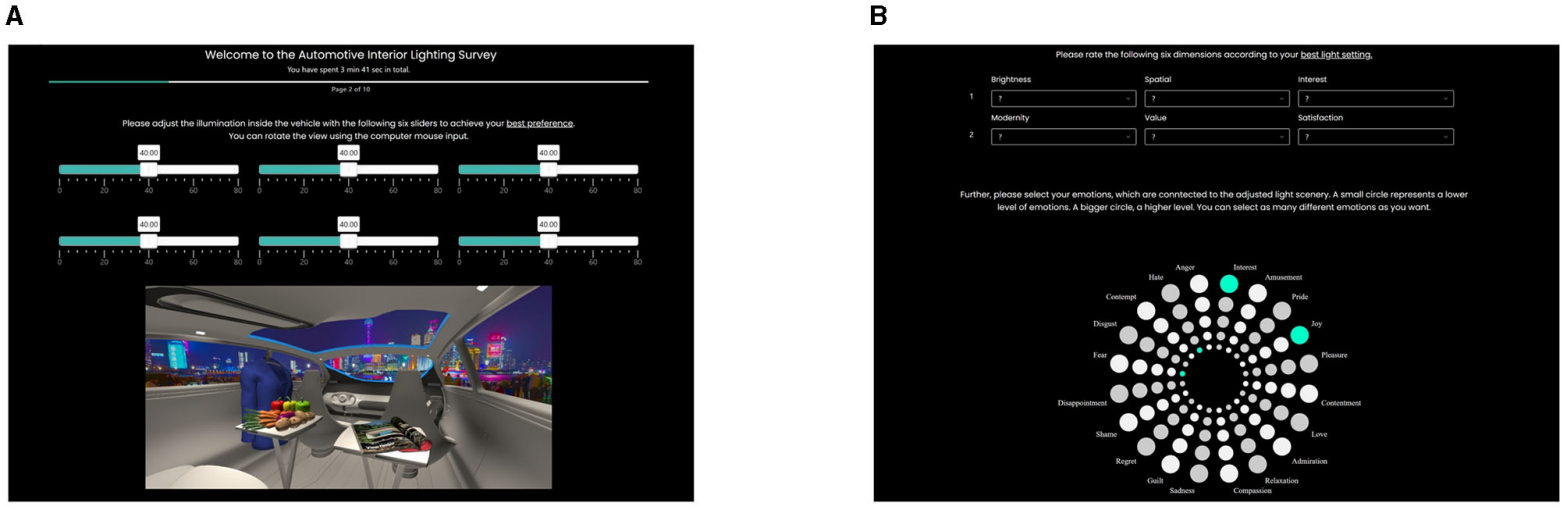
**(A)** In-vehicle 360°-night scene in Shanghai with unlabeled sliders to change hue as CCT, chroma, and lightness for spot and spatial luminaires in 80 steps each. **(B)** Emotion ratings based on our investigated semantic differentials (top) and based on the Geneva Emotion Wheel (down) are segmented into five levels and presented as different-sized circles. Green marked circles are the selected levels of emotions.

To evaluate whether favored or unfavored illumination settings can be decoded via brain activity, we first created adequate visual stimulation images. For that, we used a similar study setting as in our previous research (Weirich et al., 2022b): We arbitrarily presented four driving scenes to each subject, which were located in (a) a sunny city, (b) a dim forest, (c) a sunny countryside, or (d) the night of Shanghai, separated between a favorable and unfavorable session. Since we presented the impression as a highly immersive 360° image on a 25-inch LED-Lit screen (Dell U2518DR) operated at 60 Hz, the subjects were able to rotate the camera view within the second row of the vehicle. Furthermore, we placed six unlabeled sliders to adjust chroma, hue, and lightness for either centered focused spotlights (Lu1) or widely spread room-filling luminaires (Lu2). The slider description was missing to prevent external bias effects either from the view of long-time thoughts about their meanings or from potential preferences to adjust one slider only. All sliders should be equally recognized and operated. Since we applied white light settings only, the changes in hue were limited to step sizes located at the Planckian curve. For that, we varied the CCT from 1,000 K, a reddish warm white, to 33,100 K, a high blueish cold white. The step size was nonlinearly distributed, achieving a smaller step size at lower and a larger step size at higher CCTs. For chroma, we took 1/3 of *C*_0_ as the start value calculated from the on-plank CCT value and varied this value inside CAM16 until we achieved three times *C*_0_ as the closing value. The step size was linear since we were calculating in CAM16. For lightness, we varied between zero, where the light was turned off, and the brightest level until the displayed scene was not over-illuminated. That means, even for the brightest setting, all scene details could still be identified. For all three dimensions, we had 80 steps for adaptation using the mentioned sliders, shown in the top row in Figure 2A. The task for the study participants was to vary all six sliders until they reached a level of most preferred or most disliked in-vehicle lighting. This task was completed for each of the four external scenes. The order of the external scenes arbitrarily varied among the study participants.

Then, we asked the study participants to rate their favored and unfavored settings. We used two different metrics to measure their level of emotions. First, we used the same six fields of semantic differentials rated by a 6-point scale, as described in detail in our previous study (Weirich et al., 2022b), separated between evaluative and in-vehicle perceptions. For the evaluative domain, we set brightness, spatial, and interest, and for the in-vehicle domain, we set modernity, value, and satisfaction as perceptional dimensions. Second, to get a deeper understanding of actual emotions, we used the Geneva Emotion Wheel (GEW) (Scherer et al., 2013). We adapted the analog version to a digital one to add five circles in different sizes representing the level of emotions: smaller circles representing a smaller level and vice-versa. We also deleted the possibility of adding another subjective feeling, which was originally placed in the center of the wheel. Each subject had to rate each emotion facet. The last part of this session gave the subjects the possibility to write down their own comments, thoughts, or suggestions about this study session itself. During the answering of all questions in this session, we recorded participants' gaze data using an eye tracker (Tobii X-120 Pro) at 120 Hz to better understand which part of the displayed vehicle scene was important for their final favorable or unfavorable decision. Figure 2 summarizes and shows the session setting illustrated with the Shanghai night driving scene.

### 3.2. Session 2: cortical emotion relations based on displayed images

In the second session, we used 16-channel measurement equipment with a 3D-printed headset (OpenBCI, Ultracortex MarkIV, Open BCI, n.d.) to record EEG signals with a sample frequency of 1,000 Hz. Since we were recording with active dry electrodes (ThinkPulse, Active Electrodes), it was necessary to ensure that the electrodes were positioned suitably for noise reduction. Besides the measurement standards (Odom et al., 2016), to track electrode-skin impedance values, we defined our own valid recording parameters based on a 1-s mean value and the standard deviation and ratio of both, which can be stated as a signal-to-noise ratio (Smith, 1997). Upper and lower limits of these were defined based on an extensive pre-study.

As reviewed, frontal left and right hemisphere electrode positions are significant for identifying positive and negative emotions. Therefore, we selected the inion (IZ) to F3 for the left-side channel and IZ–F4 for the right-side channel. Finally, we added IZ–OZ for comparative cortical activity measurement. We set the left ear lobe, location A1, as the ground electrode. Electrode positions were defined according to the 10-10 international standard for electrode placements (Acharya et al., 2016).

The evoked EEG time window was set as *t*_min_ = 0.0 s and *t*_max_ = 0.5 s after stimulus onset, and single epochs were grand averaged between all eight subjects. Each epoch's rejection criterion was set to 100 μV based on several pre-study investigations with our recording equipment and based on a common understanding that the P300 amplitude might be bigger than the P100 peak (Ladd-Parada et al., 2014).

Furthermore, the EEG raw data were electronically filtered with 50 Hz and 60 Hz notch filters to reduce the electrical power noise, and a bandpass filter was applied between 3 and 45 Hz. Since in China, the powerline signal operates at 50 Hz, only a notch filter at 50 Hz might be sufficient. To be on the safe side and considering that there are no sharp transitions between the passing and blocking frequencies, we applied both notch filters with a second order. In addition, the bandpass filter frequencies were set to 3 and 45 Hz with a second-order filter for gentle filtering and to prevent overshoot or edge effects in the time domain. This range is also commonly used for brain–computer interface applications (Renard et al., 2010). Artifact removal was performed in two steps: first, visual inspection of EEG data, and second, based on the applied epoch rejection criterion at 100 μV. The baseline was corrected at the stimulus and epoch starting points, set as 0 s. Baseline and epoching procedures were performed using the Python library MNE (Gramfort et al., 2013).

As an extension of our last study (Weirich et al., 2023), which we applied in this study, we compared EEG activity between the already investigated less emotional baseline and strong emotional benchmark settings with the abovementioned four external driving scenes and their good and bad in-vehicle lighting settings. For stimulus presentation, we used 20 positive and negatively correlated images from the Geneva Affective Picture Database (GAPED) (Dan-Glauser and Scherer, 2011) to define our baseline and benchmark stimulus levels. Neutral images were characterized primarily by images of buildings, stairs, desks, or empty streets. Strong positive images showed vacation islands, sunny beaches, smiling faces, or colorful landscapes. Strong negative images consisted of snakes, spiders, or violations of animals or humans. The in-vehicle lighting stimuli were created as described in Section 3.1.

**Figure 3:**
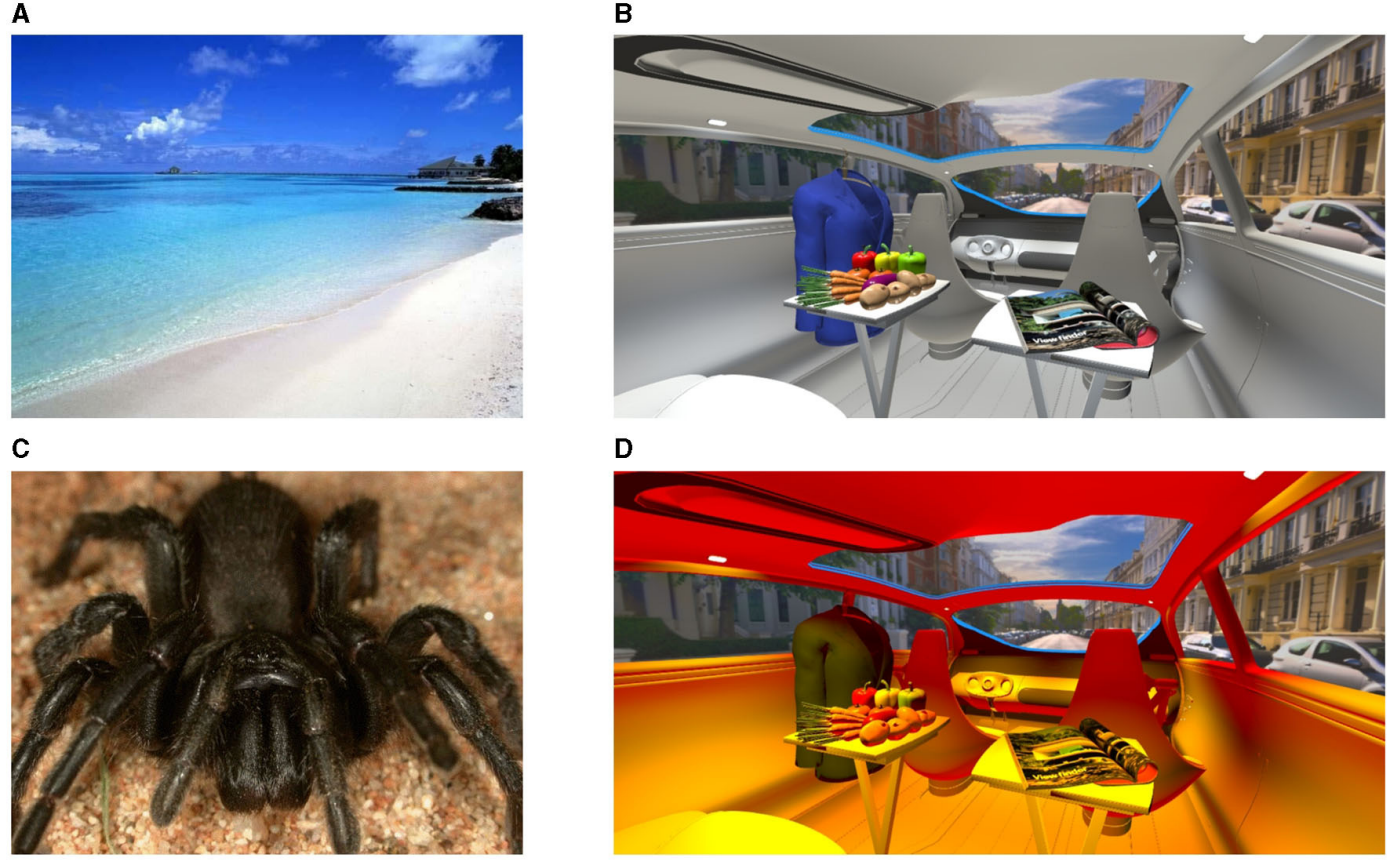
**(A, C)** Images arbitrarily selected out of the GAPED (Dan-Glauser and Scherer, 2011): **(A)** A positive and **(C)** a negative emotional image. **(B, D)** Examples from Session 2 for a favored **(B)** and an unfavored **(D)** illumination setting. **(A, C)** and **(B, D)** are also examples of a paired visual stimulation group.

As previously reviewed, we focused on later positive potential (LPP) changes, such as those occurring 300–500 ms after the stimulus onset. The reason for this is that starting with P200, early emotional arousal was measured, and P300 is characteristic of voluntary attention. LPP is connected more with motivational relevance (Righi et al., 2017). In the present study, we focused more on early and initial emotional triggering induced by in-vehicle lighting than on longer emotional relevance. For that, we set the stimulus duration to 500 ms. That means we set 30.771 frames as our stimulus duration using a 60 Hz screen frequency. We also set the stimulus repetition rate to 400, leading to a total session time of 7.1 min, including a short instruction. The stimulus protocol was as follows: First, we presented the emotional baseline defined by paired neutral images, each for 30.771 frames duration. Second, we randomly selected a pair of favored and unfavored luminaires out of one of the four driving scenes, which was defined as described in Section 3.1. This means that we started to select an arbitrary driving scene such as the night scene in Shanghai. Then, we presented paired arbitrary positive and negative created in-vehicle light settings. Consequently, during one constant driving scene session, only the in-vehicle lights were changed randomly between all eight positive and negative settings. Randomization was performed using the built-in random function in the applied stimulus presentation software Psychopy (Peirce et al., 2019). For that, all in-vehicle images separated between good and bad illumination were saved in a database. For each iteration, Psychopy randomly selected one good and one bad in-vehicle lighting setting. After all the defined settings were presented once, a second round was started by selecting a random image for the starting point. Finally, we showed strong positive and negative images as emotional benchmark stimuli, again paired. An example of highly positively and negatively correlated images of the GAPED is shown in Figures 3A, C, and favored and unfavored lighting settings from the sun-city scene are illustrated in Figures 3B, D.

## 4. Results

We investigated five women and three men with an average age class between 25 and 34 years. All were healthy with a normal 20/20 vision acuity level, had no color vision deficits, and took no caffeine or medications. In the following section, we present the results of the preference session.

### 4.1. In-vehicle lighting preferences

As mentioned in Section 3.1, we recorded gaze data at 120 Hz and asked the study participants to adjust six unlabeled sliders to achieve a good or bad in-vehicle lighting scene at four different locations and time settings, including their emotional corresponds. Statistical analysis was performed by comparing settings for bad and good lighting groups. Since both settings were performed by the same study participants and values were ordinal scaled, the Wilcoxon signed-rank test was conducted. The significance level α was set to 0.05. No corrections for multiple comparisons, such as the Bonferroni correction, were applied. The effect size was calculated using the coefficient *r* = |z|/√n, as recommended (Fritz et al., 2012).

**Figure 4:**
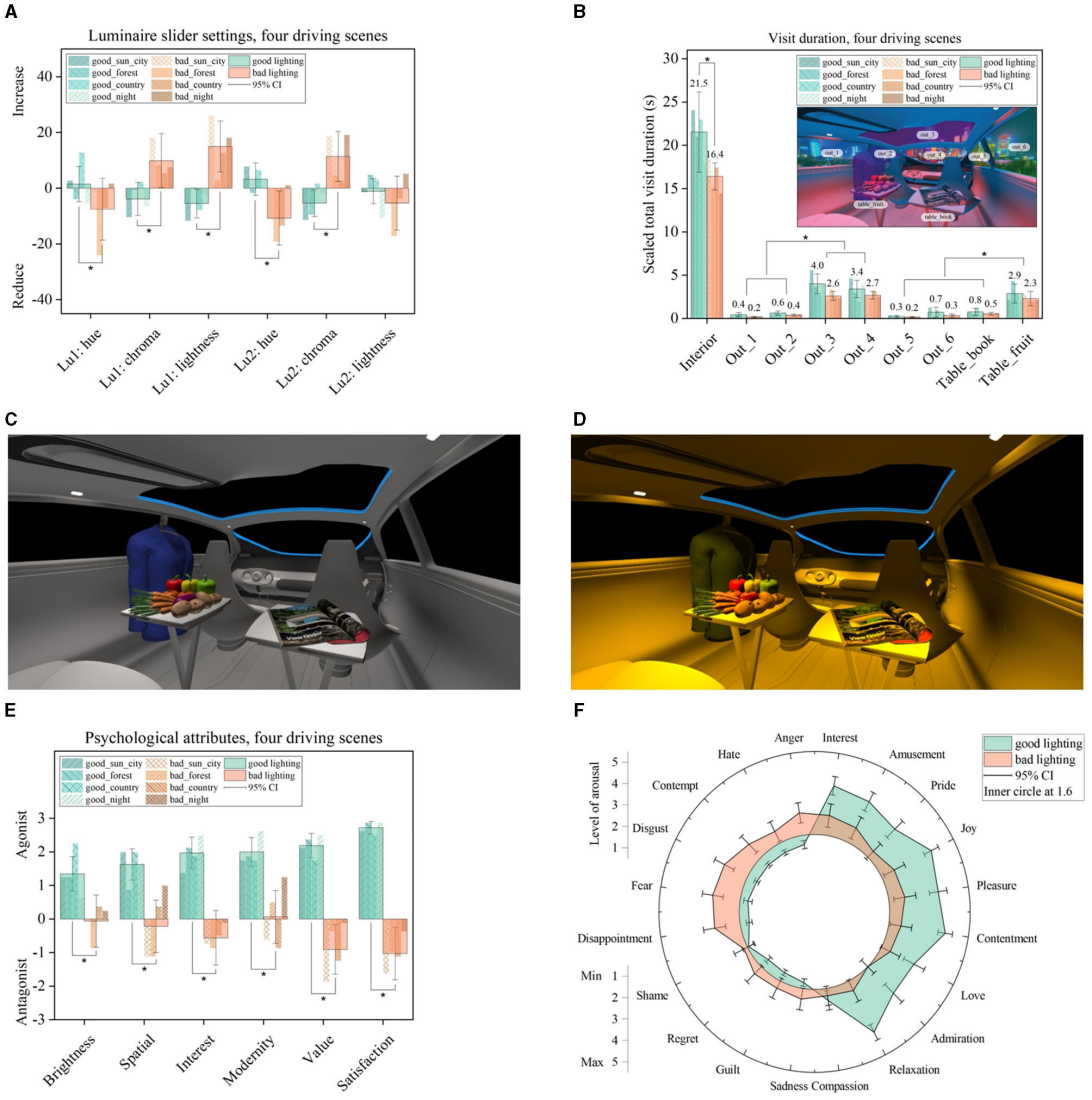
**(A)** Best and worst slider settings separated and averaged within the four driving scenes shown. Besides the hue attribute of the spot luminaire Lu1 and the lightness attribute of the spatially spread luminaire Lu2, other perceptional dimensions showed a significant difference between good and bad settings, as marked (*p < 0.05). **(B)** Gaze data were evaluated as the total visit duration separated between the interior and exterior areas of interest. Furthermore, the fruit table with the blue jacket had a higher visit duration than the magazine table (*p < 0.05). **(C)** Averaged example of good lighting setting compared to **(D)** bad lighting settings. **(E, F)** Emotional ratings according to the adjusted light scene.

First, we summarize the averaged slider value for good or bad lighting settings in Figure 4A. Since we changed chroma and lightness based on a CCT as the hue, a zero hue value represented a CCT of 4,800 K, with zero chroma changes at the Planckian locus at an average screen brightness, represented by zero values for all three dimensions, as illustrated in Figure 4A. We found highly significant differences (*p* < 0.05) between good and bad settings. For that, the four external driving scenes were combined and evaluated for the eight subjects, leading to a sample size of *n* = 4 × 8 = 32. Since *n* > 30, the asymptotic *p*-value was reported. For the spotlight setting, Lu1 differences were observed in the dimensions of chroma (*z* = −3.909, *p* = 9.25 × 10^−5^, *n* = 32, and *r* = 0.69), lightness (*z* = −4.658, *p* = 3.18 × 10^−6^, *n* = 32, and *r* = 0.82), and hue (*z* = 3.012, *p* = 2.59 × 10^−3^, *n* = 32, and *r* = 0.53). Furthermore, for the spatial luminaire Lu2, these significant differences could be identified in the dimensions of hue (*z* = 4.059, *p* = 4.90 × 10^−5^, *n* = 32, and *r* = 0.71) and chroma (*z* = −4.367, *p* = 1.25 × 10^−5^, *n* = 32, and *r* = 0.77). Since all effect sizes were larger than 0.5, an observed strong effect size was found following Cohen (1988).

In summary, a good lighting setting has a neutral white CCT with a more unsaturated tiny darker tendency; compare Figure 4A. That means good luminaire settings could be found in the intermediate white area, as defined between 3,300 and 5,300 K (EN 12464-1, 2011), located closer to the white point. Contrarily, the eight study participants defined a bad lighting setting as a higher saturated warm–reddish hue and a high brightness setting, which was primarily applied at the spotlight luminaire Lu1. That means bad luminaire settings are often located in the warmer white area, 3,000 K and below (EN 12464-1, 2011), and they are located farther away from the white point. No brightness changes between good and bad lighting settings were applied for the second luminaire Lu2, which controlled the spatial illumination.

To represent this observation for good and bad illumination, we chose the biggest difference from the initial zero level inside the calculated confidence interval for hue, chroma, and lightness. The results are shown in Figure 4C for a good setting and in Figure 4D for a bad setting, both with a black background. Second, the total visit duration was calculated from the raw gaze data and separated between the vehicle's interior and exterior areas. Furthermore, it was evaluated at special areas of interest, which consisted of first, a table with a colorful magazine and second, a mix of fruits with a blueish jacket, as in-vehicle eye-catching approaches shown in Figure 4B. Since we found a significant shorter duration within the bad lighting session, we equalized the total gaze recording within the bad and good lighting adjustments by scaling the bad gaze recordings with 1.35. Furthermore, each vehicle window was evaluated separately and labeled out_*x*, with *x* as the window index. Statistical analysis was performed by performing a *t*-test for dependent samples since the gaze data was rationally scaled. The correlation coefficient *r* was calculated as *r* = √(*t*^2^/(*t*^2^+*df* )) to calculate the effect size (Fritz et al., 2012). Within these areas, the central windows named out_3 ($\bar{t}$ = −8.819, $\bar{p}$ = 3.07 × 10^−12^, *n* = 64, and $\bar{r}$ = 0.74) and out_4 ($\bar{t}$ = −8.817, $\bar{p}$ = 2.18 × 10^−12^, *n* = 64, and $\bar{r}$ = 0.74) with the in-vehicle fruit table ($\bar{t}$ = −5.316, $\bar{p}$ = 4.93 × 10^−6^, and *n* = 64, $\bar{r}$ = 0.55) received the highest attention compared to the other selected areas evaluated by combining the ratings for good and bad lighting sessions, as marked in Figure 4B, and averaging the resulting statistics. As calculated, all effect sizes showed a strong effect with *r* > 0.50. This means that the two central vehicle windows were highly correlated to the external scene, and the fruit table with the blueish jacket was highly connected to the attention of the in-vehicle scene, which further underlines the importance of using colorful objects inside a scene rating compared to blank spaces. Furthermore, we found a significantly shorter visit duration for bad light settings than for good light settings (*t* = 2.272, *p* = 0.03, *n* = 32, and *r* = 0.38), with a medium effect size calculated based on the remaining vehicle interior, named the interior area, as shown in Figure 4B. This observation could be generally explained by the fact that setting up a satisfied level of illumination requires more iteration loops than setting up a task level just to set bad lighting. That means there are many possibilities to achieve a bad setting but only a few to achieve a good one. Based on the collection of gaze data, the left-positioned colorful fruit table including the blueish jacket, the central glass roof, and the vehicle windshield collected the highest visual attention level and were, therefore, strongly connected to visual preference ratings and in-vehicle lighting adjustments: People tend to look longer at objects that they like, as stated in the Section 2.

Figures 4E, F shows the emotional ratings with a clear positive tendency connected with the best lighting settings. For statistical analysis, we conducted the asymptotic Wilcoxon signed-rank test based on ordinal scaled values. Within the semantic differentials evaluated at negative settings, the strongest emotional impact was found in the dimensions of interest (*z* = 4.666, *p* = 3.05 × 10^−6^, *n* = 32, and *r* = 0.82), value (*z* = 4.833, *p* = 1.34 × 10^−6^, *n* = 32, and *r* = 0.85), and satisfaction (*z* = −4.626, *p* = 3.72 × 10^−6^, *n* = 32, and *r* = 0.81), matching with a high level of disappointment, fear, and disgust identified in the emotion wheel. For brightness (*z* = 4.245, *p* = 2.18 × 10^−5^, *n* = 32, and *r* = 0.75), spatial (*z* = 4.499, *p* = 6.82 × 10^−6^, and *n* = 32, *r* = 0.79), and modernity (*z* = 4.572, *p* = 4.81 × 10^−6^, and *n* = 32, *r* = 0.80), the impact for a bad light setting was less, which was also supported by a higher level of interest, amusement, pleasure, and contentment. All effect sizes showed a strong effect level. That means a worse lighting setting was still able to evoke positive emotions, at least partly. On the contrary, a good lighting setting could not support negative feelings.

### 4.2. Cortical emotion relations

**Figure 5:**
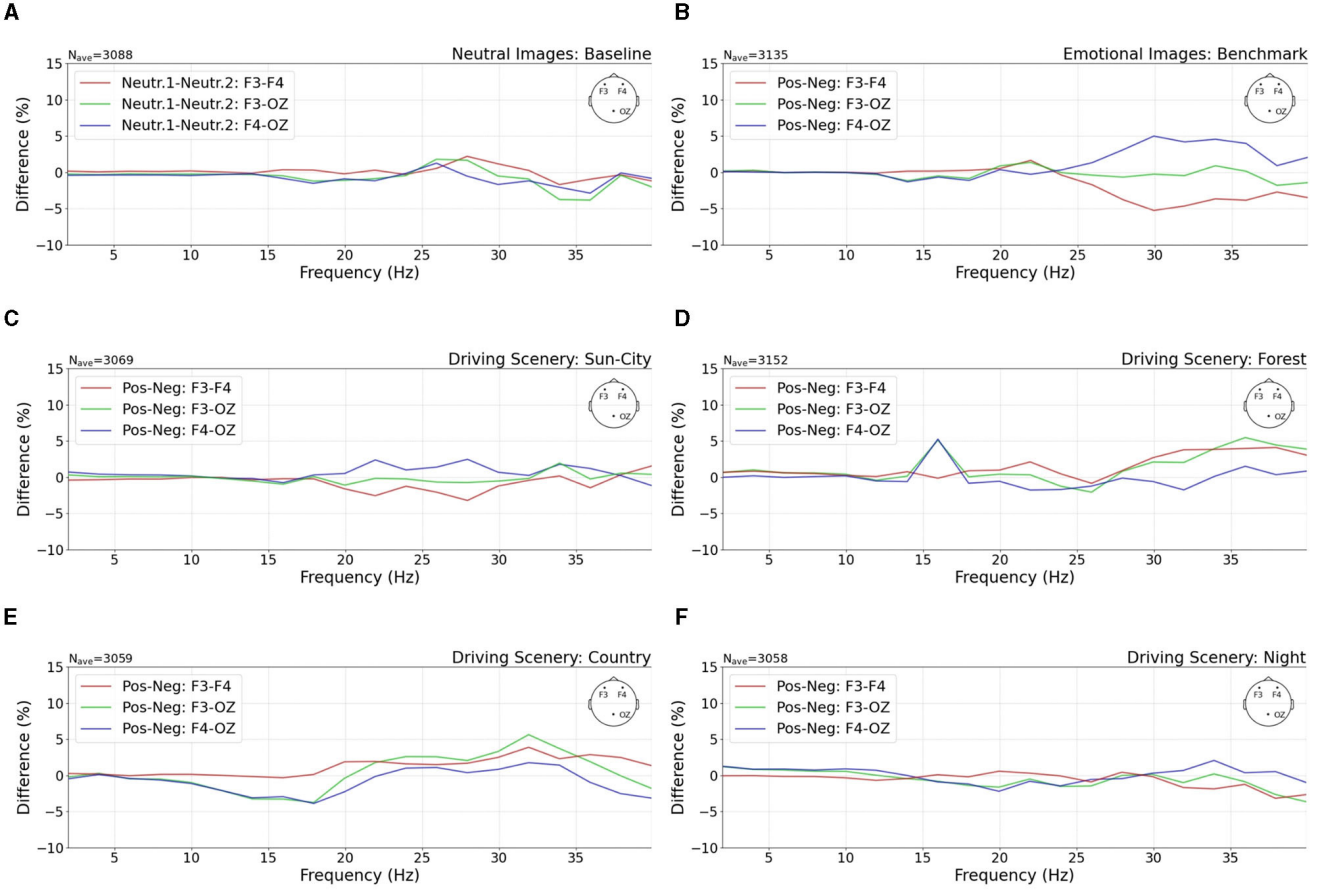
Separation between three investigated channel differences of F3–F4, F3–OZ, and F4–OZ. The power spectral density (PSD) differences between **(A)** neutral stimulus as the baseline and **(B)** positive and negative stimuli as the benchmark are shown. The presented PSD shows a high relation with evoked emotions. That means the right electrode position F4 exhibits higher activation during the negative benchmark setting, as shown by the red drop and blue peak. **(C–F)** Emotions evoked in four driving scenes. Especially in the sun-city scene, a similar channel difference is shown as in the benchmark study setting. **(A, B)** Were taken from our previous research (Weirich et al., 2023).

To get a deeper insight into the differences in cortical activity, we calculated the power spectral density (PSD) for the benchmark, the baseline, and four driving scenes, as shown in Figure 5. For that, the raw EEG data were first epoched based on the 500 ms stimulus window. Next, a grand average was calculated based on all single epochs and within all eight study participants. This averaged time-domain signal was then transferred to the frequency domain using the multitaper method (Slepian, 1978). Next, the difference in the power frequency distribution was calculated between electrode locations F3–F4, F3–OZ, and F4–OZ and presented positive and negative emotional stimuli. During the baseline condition, two paired neutral images were presented. The cortical activity between these two stimuli was calculated and described as neutral stimulus 1 and neutral stimulus 2, as shown in Figure 5A.

We found a higher right hemisphere activity connected to negative emotions, illustrated as a red drop and blueish peak in Figure 5B. Both phenomena were missing during the baseline condition, as shown in Figure 5A. During this session, all three recorded EEG channels followed the same pattern.

The frequency distribution of the three EEG channels in the four investigated driving scenes is presented in Figures 5C–F. In the figure, it is shown that for the sun-city driving scene, a similar higher right hemisphere activity was observed, which was less pronounced but followed the same lateral activity distribution as our defined benchmark stimuli; compare Figure 5B. This means that the arbitrary image presentation of paired good and bad luminaire settings was able to evoke similar cortical activities as recorded during high emotional stimulation, especially in the sun-city scene. At first, when visually compared, the other three driving scenes showed no congruent behavior at the investigated power spectral distribution compared to the baseline or benchmark setting. That means we probably found little evidence that subjective emotional ratings and emotionally correlated presented light settings might be connected and described by cortical signal changes, especially in the frontal hemispheres.

### 4.3. Cortical signal classification

**Figure 6:**
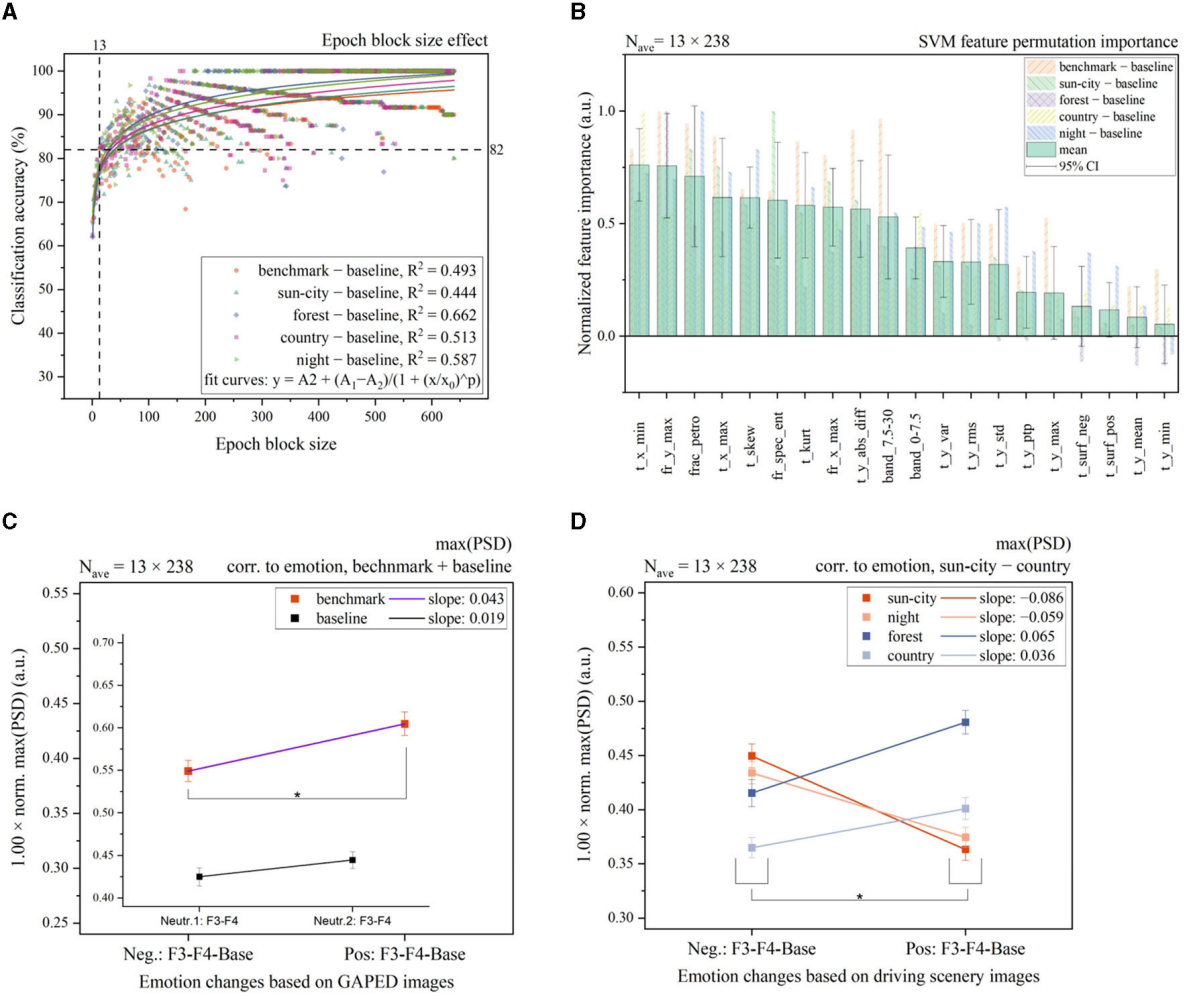
**(A)** SVM classification accuracy based on different epoch block sizes. After averaging 13 single epoch elements, a classification accuracy of 82% was observed in the benchmark baseline setting. Similar classification patterns were observed between all four driving scenes. **(B)** Normalized permutation importance calculated for each EEG signal feature. Feature names are defined by time t, frequency fr, or fractional dimensions frac. Next, the axis and applied function are written. The time points at the smallest amplitude (t_x_min) and the PSD maximum (fr_y_max) were identified as features with the highest importance. The lowest amplitude in the time domain (t_y_min) had the lowest importance. Negative values induce worse classification performance if a specific feature is used. **(C, D)** By applying the maximum PSD cortical feature with a block size of 13 × 238, a high significant difference (*p < 0.05) between the benchmark images and all four driving scenes, as marked, was observed. Slope changes between sun-city + night compared with forest + country scenes indicate a further emotional offset, independent of the light settings. **(C)** Was taken from our previous research (Weirich et al., 2023). **(B)** Feature description from left to right: Time point at the minimum amplitude, the maximum of PSD, Petrosian fractal dimension, time point at the maximum amplitude, skew, spectral entropy, kurtosis, the frequency at the maximum of PSD, the sum of absolute differences, relative band power between 7.5 and 30 Hz, relative band power between 0 and 7.5 Hz, amplitude variance in time, root mean square of amplitude in time, the standard deviation in time, the peak-to-peak ratio in time, maximal amplitude in time, negative area in time with abscissa as the reference, positive area in time with abscissa as the reference, amplitude mean value in time, and time point at minimum amplitude. Relative band power was divided into two sections for initial investigation, combining first, the delta and theta waves (0–7.5 Hz) and second, the alpha, beta, and gamma waves (7.5–30 Hz).

To further investigate this observation that there might be a correlation between good and bad in-vehicle lighting and PSD, we first applied a support vector machine (SVM) classification based on a radial basis function as the kernel. The SVM classification performance was first evaluated based on different epoch block sizes, as shown in Figure 6A. Our selection criterion was defined to obtain a high classification accuracy at a small epoch block size to maintain a high number of remaining samples for statistical comparison. For the classification procedure, we defined 20 classification features from time, frequency, and fractional space, as shown in Figure 6B. In this figure, the time point at the lowest amplitude (t_x_min) and the maximum PSD amplitude (fr_y_max) had the highest feature importance ratings within the benchmark setting and four driving scenes subtracted by the baseline setting. Hereafter, we focus on the interpretation based on the identified PSD numerics alone, since this observation is congruent with previously investigated frontal asymmetrical cortical activity in the frequency domain as a highly correlated index for identifying emotional behavior (Ahern and Schwartz, 1985; Byrne et al., 2022; Weirich et al., 2023). This means that PSD might be important for strong emotional stimuli and also in the context of in-vehicle lighting preferences. The PSD represents the power distribution of the EEG signal in the frequency domain. Therefore, the presented power per frequency bands can be calculated, such as for the alpha waves, or global metrics, such as maximum and minimum, in a more general context.

For the calculation of EEG features and the classification procedure, we followed the identified effect observed in Figure 5. This means that the single epochs in the time domain of the raw EEG signal were first subtracted between the left F3 and right F4 electrode positions only. Signals from the OZ location were not considered for feature calculation. Next, for the emotional benchmark and driving scenes, a further subtraction from the neutral emotion baseline session was performed. For reference, the resulting epoch signal for the benchmark session can be defined as follows: epochs (benchmark) = epochs (benchmark, F3)–epochs (benchmark, F4)–epochs (baseline) with epochs (baseline) = epochs (baseline, F3)–epochs (baseline, F4). Since the total recorded epoch numbers were unequal based on the applied rejection criterion, the epoch block with smaller epoch numbers was considered the limit for subtraction. Finally, 13 epochs were averaged, and based on this evoked signal, time and frequency features were calculated; compare Figure 6A. This means, we only evaluated relative changes in evoked potentials between the left and right hemispheres and baseline settings. No absolute EEG feature values based on evoked EEG data were calculated.

EEG features are described by their mathematical function in the description in Figure 6. Their names follow the structure to set the first *t* as time, *fr* as frequency, *frac* as fractional dimension, and *band* as relative band power. Next, the connected axis is written, and finally, the applied function is written, such as *min* stands for minimum. For references, t_x_min stands for the time point at the amplitude minimum in the time domain. Moreover, t_y_min is the value of the amplitude minimum in the time domain.

In the second step, we performed a *t*-test for dependent samples to test for paired significant calculations to prove the validity of the PSD maximum. We set the significant level α = 0.05 and normalized sign conserving the PSD maximum between −1 and 1, calculated employing Welch's method (Welch, 1967). The difference between positive and negative settings based on the benchmark and baseline conditions is shown in Figure 6C. The analysis showed a small effect size (*t* = 3.351, *p* = 9.36 × 10^−4^, *n* = 238, and *r* = 0.21) for the benchmark session and no significant effect for the baseline session. Here, it must be emphasized that for the baseline condition, we arbitrarily paired two neutral images only, as shown in the inner layer in Figure 6C. Furthermore, the emotional rating of each of the four in-vehicle lighting scenes is shown in Figure 6D. To be able to compare the baseline, benchmark, and four driving scenes, the epoch block size of 13 was fixed based on our selection criterion, compare Figure 6A, to obtain 238 samples for statistical evaluation. The identified feature with the maximum PSD could significantly separate positive and negative in-vehicle lighting settings within all four driving scenes. We observed that in two of the four scenes, the forest (*t* = 4.025, *p* = 7.66 × 10^−5^, *n* = 238, and *r* = 0.25) and the countryside (*t* = 2.749, *p* = 6.43 × 10^−3^, *n* = 238, and *r* = 0.17), with a small effect size, followed the trendline for a higher right cortical activity during increased negative feelings. For sun-city (*t* = −5.982, *p* = 8.11 × 10^−9^, *n* = 238, and *r* = 0.36) with a medium effect size and night (*t* = −4.487, *p* = 1.13 × 10^−5^, *n* = 238, and *r* = 0.27) with a small effect size, this effect was inversed. That means there might be an additional emotional offset produced by higher interesting external scenes compared to a monotonous forest or countryside setting, which contradicts the evoked emotional changes created by light stimuli. Consequently, cortical activities were evoked based on bad lighting, which follows the trend of positive emotions.

To further elaborate that the investigated single EEG feature, the PSD maximum, plays an important role in identifying positive and negative emotions in the context of in-vehicle lighting, the identified frequencies at the maximum power were statistically analyzed based on the defined feature named fr_x_max. Statistical analysis was performed using a *t*-test for dependent samples. No significant differences could be found during the benchmark setting and within the four external driving scenes based on 13 averaged epochs. Relative changes of the frequencies at the PSD maximum, which were evoked and transferred to the frequency domain, were on average 3.11 ± 0.49 Hz for the positive and 3.06 ± 0.50 Hz for the negative stimuli during the benchmark settings. Over all vehicle driving settings, the relative frequency varied on average 2.60 ± 0.19 Hz for the positive emotions and 2.69 ± 0.19 Hz for the negative emotions. This means that there were neglectable relative frequency changes observed, as also identified by the abovementioned missing significances in the *t*-test.

## 5. Discussion

We applied our identified cortical EEG feature, the PSD maximum (Weirich et al., 2023), which can also be stated as a more general index compared to the frontal alpha asymmetry (FAA) (Ahern and Schwartz, 1985; Byrne et al., 2022), which only takes the alpha waves (7.5–12 Hz) into account in the context of in-vehicle lighting scene preferences. We compared the results with emotional ratings and gaze data recorded via eye-tracking. First, we decided to take the total visit duration, defined as the time between the first and last fixation on an area of interest (AOI), as our key index calculated by gaze data, which was suggested in an extensive review about the correlation between eye-tracking metrics and cognitive or emotional processes (Skaramagkas et al., 2023). They identified fixation duration, the number of fixations, total fixation time, saccade amplitude, and the number of saccades as strong indices connected with visual attention. Moreover, fixation duration, the first fixation probability, blink rate, and pupil size were correlated with an increase in emotional arousal. Combining both, only fixation duration could identify both dimensions, which, integrated over time and AOI, represents the total visit duration per AOI. Within the investigated AOIs, the central vehicle windows and the left side-orientated colorful fruit table together with the blueish jacket received significantly longer visits during the best and worst adaption processes, as shown in Figure 4B. For the central vehicle windows representing the external driving scene, this phenomenon is widely understood in the literature as “central bias”. Physiologically roughly explained, a center starting point is highly favorable for following oculomotor saccade activations for scanning additional image areas (Tatler, 2007). The fruit table located at the left side in-vehicle area collected significantly more attention than the front right-side table with the colorful magazine. This means that daily colorful objects are highly associated with preference ratings and should be applied in a related scene for preference ratings.

Second, it is shown in Figures 4A, C that the hue for the best lighting setting between spot- and spatial luminaires did not vary, which clearly contradicts our previous finding (Weirich et al., 2022b): We found that the best lighting setting should be a mix of lower and higher CCTs with the same scene setup. One explanation for this could be, first, the smaller sample size within this study when compared to our previous study. Second, in our previous study, we rated preselected luminaire settings. In the present study, we allowed participants to freely choose between various possibilities. Anyway, our aim within this study was not to define the best setting. Rather, we focused on the root cause to identify why one specific luminaire vector consisting of hue, chroma, and lightness is able to achieve a supporting or contradicting subjective evaluation.

The results of the identified semantic differentials and emotion wheel, Figures 4E, F, are congruent in terms of satisfaction, value, and interests, which are clearly worse for bad applied lighting. Moreover, the identified bad lighting settings were still able to evoke, at least to some extent, positive emotions in the fields of joy, pleasure, and contentment. Actually, how the quality of lighting can be defined is still under debate. One direction is to focus on the combination of space, user task, and applied lighting (Allan et al., 2019). However, to what extent and with which metrics subjective evaluations should contribute to this very general approach has not yet been decided (CIE, 2014).

Furthermore, the decoding of positive and negative emotions was congruent with the literature (Ahern and Schwartz, 1985): A higher level of maximum PSD was observed in the right hemisphere for negatively correlated emotions; compare Figures 5B, 6C. Here, this frontal asymmetry was especially observed in the gamma frequency band; it was observed less in the beta band and was nearly negligible in the alpha band. Moreover, a contradiction was observed between the presented external driving scenes for the sun-city and night settings, as shown in Figure 6D. Here, a subjectively rated negative in-vehicle light setting created a cortical activity that significantly correlated with a higher level of positive emotions. This contradiction between subjectively rated scale settings and measured cortical activity could be explained based on the viewing content. Both scenes can be stated as highly interesting compared to the monotonous countryside or forest setting. That means here, we probably biased the evoked illumination-based emotions with interesting stimuli. The same separation between external interesting and monotonous scenes was also identified in the second part of this mini-series (Weirich et al., 2022b). This phenomenon gives further research a great opportunity for deeper insights, perhaps by also combining several EEG features, as shown in Figure 6B, to optimize the classification accuracy in two or more emotional settings. One example of that might be the further subdivision of the applied relative band power. In this initial investigation, we separated the bands only between 0–7.5 Hz and 7.5–30 Hz, considering that for frequencies below 3 Hz, the amplitude is affected by the described bandpass filter, as mentioned in Section 3.2. As identified by the permutation feature importance analysis, the 7.5–30 Hz group achieved a higher level of importance than the second group. This means follow-up studies can separate the higher frequency group by narrowing the important frequency ranges.

Furthermore, it was reviewed that there might be a neuronal connection between P300 and LPP, and both responses are related more to the significance of the stimulus than to the stimulus itself (Hajcak and Foti, 2020). In this extensive review, LPPs are defined starting at 300 ms after stimulus onset in ERPs, and the signal is maximized at the centroparietal electrodes such as CPz. In emotional responses, only a slight difference between unpleasant and pleasant emotional images was recorded, but a large signal difference between both emotional settings and neutral stimuli was found. The P300, elicited by an oddball task, was further increased if the target was connected to emotions (Schupp et al., 2007). This means that the more important, the more personally significant, or the more memorable a stimulus is perceived to be, the greater the cortical activity (Donchin, 1981). Taking these findings in the context of the presented study, the following conclusions can be stated in the context of neuroaesthetics. First, a time window of 500 ms, or alternatively 200–350 ms, is sufficient (Schupp et al., 2007), after stimulus onset for recording cortical activities in the content of less emotional image ratings by focusing on either P300 or LPPs, since both potential changes are actually connected to each other and are represented in a similar time window. This means that in-vehicle lighting preferences alone, without direct emotional stimuli, can be decoded by cortical activities, and this presents a novel finding in the field of neuroaesthetics. Second, since both potentials are connected to each other, our introduced approach investigating defined EEG signal features in the time and frequency domain, compared to focusing on potential changes in ERP-located time windows alone, might create the necessary difference in investigating cortical activities during an emotional task for modeling and predictions. Within this approach, the same single EEG feature, the maximum of PSD was able to significantly classify strong positive and negative emotional images and less emotional in-vehicle preferences based on lighting variations. However, we present the initial findings of this study. Further deeper research is necessary to build upon this new target by counterbalancing the impact of light itself and addressing the significant differences of single EEG signal points, such as maximum and minimum, and integrations of these such as band powers. This will also help understand deeper neural mechanisms. Finally, we had several limitations within our study. First, the investigated sample size of eight study participants can be stated as relatively small. However, since we primarily evaluated gaze data, recorded with 120 samples per s, and emotionally evoked potentials with 400 repetitions per study setting and subject, our statistical power was sufficient. This was primarily displayed in identified significant correlations and by following a recommendation for epoch trails within smaller subject groups targeting a significance level of 0.05 in the field of decoding color and orientation (Hajonides et al., 2021). The identified best and worst luminaire settings should be carefully judged because this was not our primary target within this study, as stated before.

## 6. Conclusion and outlook

In part three of our mini-series, we extended our method to define the best or worst in-vehicle lighting setting with cortical activity. We first identified, based on gaze data, highly correlated scene objects that are connected to subjective emotional assessments. Second, in this controlled laboratory study, we successfully applied the PSD maximum as the cortical EEG feature for positive and negative emotions in different external driving scenes with highly positively and negatively correlated luminaire settings. Two more monotonous external driving scenes congruently followed the emotional benchmarks. For the more interesting external scenes, despite the highly negative emotional rating, worse light settings were still able to create emotional cortical activity, which followed a trendline for positive settings. That means high-frequency stimulations of subjectively bad-rated luminaire settings are still able to evoke more positive or interesting associated cortical activity, which underlines the importance of further research in this combination of psychophysiological color science and neuroaesthetics. For our wider study target, to determine the optimal in-vehicle lighting setting, we found an objective measurable cortical body parameter connected to subjective illumination preference. That means now we can probably connect illumination and subjective scene preferences objectively and measurably or perhaps also in a control-loop setup.

## Data availability statement

The datasets presented in this article are not readily available because no data will be shared outside the research team. Requests to access the datasets should be directed to YL, ydlin@fudan.edu.cn.

## Ethics statement

The studies involving humans were approved by the Ethical Committee of Fudan University, Approvement Number FE23073R. The studies were conducted in accordance with the local legislation and institutional requirements. The participants provided their written informed consent to participate in this study.

## Author contributions

Conceptualization, methodology, software, formal analysis, and writing—original draft preparation: CW. Writing—review and editing: CW, YL, and TK. Supervision: YL and TK. All authors have read and agreed to the published version of the manuscript.
